# Cardiorespiratory fitness modulates prestimulus EEG microstates during a sustained attention task

**DOI:** 10.3389/fnins.2023.1188695

**Published:** 2023-06-15

**Authors:** Francesco Di Muccio, Marie Simonet, Catherine Brandner, Paolo Ruggeri, Jérôme Barral

**Affiliations:** ^1^Brain Electrophysiology Attention Movement Laboratory, Institute of Psychology, University of Lausanne, Lausanne, Switzerland; ^2^Institute of Sport Sciences, University of Lausanne, Lausanne, Switzerland

**Keywords:** sustained attention, cardiorespiratory fitness, physical activity, EEG, microstates analysis, pre-stimulus, aerobic fitness

## Abstract

Higher cardiorespiratory fitness is associated with an increased ability to perform sustained attention tasks and detect rare and unpredictable signals over prolonged periods. The electrocortical dynamics underlying this relationship were mainly investigated after visual stimulus onset in sustained attention tasks. Prestimulus electrocortical activity supporting differences in sustained attention performance according to the level of cardiorespiratory fitness have yet to be examined. Consequently, this study aimed to investigate EEG microstates 2 seconds before the stimulus onset in 65 healthy individuals aged 18–37, differing in cardiorespiratory fitness, while performing a psychomotor vigilance task. The analyses showed that a lower duration of the microstate A and a higher occurrence of the microstate D correlated with higher cardiorespiratory fitness in the prestimulus periods. In addition, increased global field power and occurrence of microstate A were associated with slower response times in the psychomotor vigilance task, while greater global explained variance, coverage, and occurrence of microstate D were linked to faster response times. Our collective findings showed that individuals with higher cardiorespiratory fitness exhibit typical electrocortical dynamics that allow them to allocate their attentional resources more efficiently when engaged in sustained attention tasks.

## 1. Introduction

The capacity to sustain attention over time is critical in many everyday activities, such as work, study, driving, leisure activities, and sports. Sustained attention is the ability to detect rare and unpredictable signals over prolonged periods (Sarter et al., 2001). Previous literature showed that the ability to sustain attention positively correlates with the cardiorespiratory fitness (CRF) level (Luque-Casado et al., 2016; Ciria et al., 2017; Sanabria et al., 2019; Di Muccio et al., 2022) which corresponds with the capacity of the circulatory and respiratory systems to deliver oxygen during prolonged physical activity (Caspersen et al., 1985).

At brain level, specific networks (i.e., the dorsal frontoparietal (DAN), default mode (DMN), and cingulo-opercular (CON) networks) support sustained attention (Esterman et al., 2013; Hilti et al., 2013; Langner and Eickhoff, 2013; Coste and Kleinschmidt, 2016; Kucyi et al., 2016). Nevertheless, functional changes in the brain that correlate with interindividual variations in CRF are poorly studied. Moreover, the mechanisms involved in brain stimulus-related readiness – which corresponds to the period preceding the stimulus onset (prestimulus) – modulated by the level of CRF is scarcely studied. In this study, we therefore investigated the relationship between the prestimulus electrocortical brain dynamic and the CRF in a sustained attention task.

Previous electroencephalographic (EEG) studies used cognitive tasks to identify temporal characteristics involved in brain processing in individuals with unequal levels of CRF. Many of these studies focused on the poststimulus period and used P3b event-related potentials (ERPs) to highlight differences in the cognitive processing of stimuli between high-and low-fit individuals. The results showed that individuals with enhanced levels of fitness or who were more active had larger P3b amplitudes and shorter P3b latencies when performing tasks requiring cognitive control (including sustained attention) than those who were less fit or active (Hillman et al., 2012; Kao et al., 2019). Interestingly, the P3b ERP component elicited during target processing correlates with changes in attentional resources (Polich, 2007). Recently, Di Muccio et al. (2022) explored the relationship between CRF and electrocortical brain processing within the time window corresponding to the P3b component. The authors found an increased activation within the posterior cingulate cortex (PPC) – a major node for the DMN – among the fittest participants. In addition, topographic analyses revealed that the supplementary motor areas (SMA) – a component of DAN (see Langner and Eickhoff, 2013) – activated earlier in high-fit participants and permitted faster response times. Even though the results of these studies are susceptible to reactive attentional processes that occur after the stimulus onset, the impact of CRF on prestimulus electrocortical activity in a sustained attention task is unknown.

Few studies have investigated the prestimulus period during sustained attention tasks using EEG methods. In a study by Minkwitz et al. (2011), higher prestimulus alpha activity over the occipital region correlated with faster response times in a sustained attention task. Based on this result, the authors concluded on modulations of vigilance state at the origin of the fluctuations of attentional performance. Similarly, Busch and VanRullen (2010) found that the detection performance for attended stimuli fluctuated with the phase of spontaneous oscillations in the θ (≈7 Hz) frequency band just before the onset of the stimulus. These results suggest that sustained attention exerts its facilitative effect on perception periodically. However, these studies did not fully exploit information from high-density EEG recordings to provide increased accuracies for describing prestimulus brain dynamics.

Due to the spontaneous nature of the brain activity within the prestimulus periods of a sustained attentional task, where stimulus appearance is unpredictable, EEG microstate analyses are ideal for investigating the relationship between prestimulus brain dynamics and CRF. EEG microstate analyses reduce the EEG signal to a time series of quasi-stable brain states, identified by a unique electrical potential topography that remains stable for 60 to 150 milliseconds before moving to another microstate (Khanna et al., 2015; Michel and Koenig, 2018). Therefore, the dynamics of brain states during prestimulus periods can be captured with a high degree of temporal accuracy. In the literature, four or more identified microstates were associated with different brain networks: auditive (A), visual (B), default mode (C), attentional (D), and salience (E) networks (Britz et al., 2010; Custo et al., 2017). Although these brain networks were identified under resting conditions, previous studies confirmed that these microstates might be linked with cognitive tasks. A study investigating the relationship between EEG microstates and mental subtraction tasks showed an increase in the prevalence of microstate D and a decrease in the prevalence of microstate C during the task compared to a state of resting (Seitzman et al., 2017). A study comparing the microstate dynamics between good and poor performers of a mental subtraction task showed that good performers had an increased prevalence of microstate D and a decreased prevalence of microstate C during the task compared to a rest state (Kim et al., 2021). Moreover, modulations of the microstates dynamics under different types of tasks or conditions have been reported, such as in visual and auditive memory tasks, sleep, breath-focused meditation, and physical exercise (Spring et al., 2017; Bréchet et al., 2019, 2020, 2021; D’Croz-Baron et al., 2021). Still, among the studies that have examined the prestimulus periods in cognitive tasks using microstates analysis, the focus was put on the emergence of perceptual awareness (Britz et al., 2009, 2014; Pitts and Britz, 2011; Spadone et al., 2020). A single study assessed EEG microstates within the prestimulus period during a sustained attention task (Zanesco et al., 2020a). The authors reported that an increased prevalence of microstate C before stimulus onset correlated with increased variability in response time (i.e., poor sustained attention performance). Overall, these studies suggest that during cognitive tasks (including sustained attention tasks), microstates C and D may represent task-negative and task-positive brain states, respectively.

The purpose of this study is to investigate the relationship between prestimulus electrocortical activity, CRF levels, and response times using the psychomotor vigilance task (PVT) (Wilkinson and Houghton, 1982). The PVT’s random single modality interstimulus intervals are well suited for EEG microstate analyses of prestimulus periods. Therefore, we leveraged the EEG dataset from previous Di Muccio et al. (2022) study. Since individuals who are more fit are expected to outperform their lower-fit counterparts during PVT (Di Muccio et al., 2022), we hypothesized that individuals with higher CRF levels and faster response times would exhibit a lower prevalence of microstate C, suggesting a reduced implication of DMN, and a higher prevalence of microstate D, suggesting a higher implication of DAN.

## 2. Materials and methods

### 2.1. Participants

The behavioral (PVT response times), cardiorespiratory, and EEG database is the same as the one used in the recent work of Di Muccio et al. (2022). A power analysis was conducted to estimate the minimum sample size required for a power level of 0.80 and a medium effect size (*f* = 0.15). This analysis indicated a minimum of 68 participants. Based on this analysis and considering the possible dropouts, 72 right-handed young adults (descriptive statistics in Table 1) with normal or corrected-to-normal vision and no history of neuropsychiatric disorders were selected. Self-screening questionnaires (PAR-Q, Physical Activity Readiness Questionnaire) assessed their ability to perform intense physical activity without risk to their health (Adams, 1999). Due to poor EEG signal quality, seven participants were excluded from the analyses. The remaining sample consisted of 65 participants (20 males; mean age = 21.9, SD = 3.6, range 18–37 years old) for the EEG analyses. All participants in this study provided informed written consent and the Cantonal Ethics Committee for Human Research (Vaud, Switzerland; protocol 2018-02107) approved the study.

**Table 1:** Descriptive statistics of participants for EEG data.

Descriptive characteristics	M	SD	Min	Max
*N* = 65 (20 males)				
**Age**	21.90	3.60	18	37
Female	20.8	2.14	18	29
Male	24.1	5.01	20	37
**VO**_**2**_ **max (ml/kg/min)**	43.90	9.85	24.10	77.30
Female	39.5	6.52	24.1	51.8
Male	54.00	8.58	35.6	77.3

Adapted from Di Muccio et al. (2022).

### 2.2. Study design

The investigation was carried out through two independent sessions. The first session was dedicated to measuring maximal oxygen consumption (VO_2_max) through an incremental effort test. In the second session, individual habitual physical activity was assessed using a self-screening questionnaire (Baecke et al., 1982) and continuous EEG data during a PVT was recorded.

### 2.3. Experimental procedure

#### 2.3.1. VO_2_max assessment

Participants performed an incremental effort test on a motor-driven treadmill according to Roecker’s protocol (Roecker et al., 2002). For details on the incremental effort test refer to the study of Di Muccio et al. (2022). To account for the existing physiological VO_2_max differences between women and men, the data were transformed – separately for each sex – in *z*-score values according to the reference standards for cardiorespiratory fitness of the Fitness Registry and the Importance of Exercise National Database (FRIEND) (Pate and Kriska, 1984; Kaminsky et al., 2015).

#### 2.3.2. Psychomotor vigilance task

The Psychopy software was used to display the PVT (adapted from, Dinges and Powell, 1985), record responses, and send markers to the EEG recording device (Peirce et al., 2019). During the PVT, participants were seated in a quiet room sheltered from electromagnetic disturbances (Faraday cage). They faced a monitor placed at 60 cm from their eyes and their head was placed on a chin rest to limit EEG artifacts due to head movements. In this version of the PVT, each trial began with a black screen for a random period (2–10 s), followed by a red dot stimulus appearing at the center of the screen for 500 millisconds, prompting the participant to press the space bar of the keyboard as fast as possible in a 1 s time window. If no response was given within one second or if a response was given before stimulus onset, the trial was considered as an error. Importantly, to obtain a faster decrease in performance, no feedback was given after each trial. The task lasted 30 min and consisted of 240 trials. No breaks were included in the task design.

As several factors like sleep/wake balance, arousal, motivation, and stress are known to influence the performance of sustained attention (Oken et al., 2006), participants were asked to spend a “normal” night’s sleep the day before the experiment. Also, they were asked to avoid all types of energy drinks and unusual amounts of nicotine or coffee just before the experiment. There was no financial compensation or rewards for the participants.

#### 2.3.3. VO_2_max and psychomotor vigilance task

In this article, we build on the behavioral results of the recent paper published by Di Muccio et al. (2022). In this paper, a multiple linear regression was used to investigate if the VO_2_max could predict response times in the PVT while controlling for age, body mass index (BMI) and chronic physical activity (Baecke total score). For details on the behavioral statistical analyses, refer to the study of Di Muccio et al. (2022).

### 2.4. EEG recording and pre-processing

A continuous EEG signal was recorded from 64 electrodes (Biosemi ActiveTwo system, Amsterdam, Netherlands) placed in accordance with the international 10–20 system location during performance of the PVT. Two additional electrodes were used as reference and ground (active CMS: common mode sense and passive DRL: driven right leg) to constitute a feedback loop for amplifier reference. Data were recorded at a sampling rate of 1,024 Hz with 24-bit A/D conversion.

Offline pre-processing was performed with Brain Vision Analyzer (version 2.1.2.327; Brain Products GmbH). Raw signals were filtered between 1 and 40 Hz using a zero-phase shift second order butterworth filter and downsampled to 128 Hz. Next, eye blinks and saccades were corrected using an independent component analysis (ICA) (Cardoso, 1998). Bad electrodes were interpolated using linear splines interpolation of adjacent electrodes (Perrin et al., 1987). Next, data were segmented into 2000 milliseconds prestimulus epochs preceding the target apparition (240 epochs per participant). Epochs containing electrical potential values exceeding ±80 μV were excluded. In addition, signals were visually inspected for residual artifacts. After the pre-processing, the mean number of remaining epochs was 225.05 ± 16.81. Epochs were then concatenated into one file per participant. Then, these concatenated epochs were processed into a continuous file, but the transition periods were marked as an artifact so that they would not be considered in cluster analyses.

### 2.5. EEG microstates analysis

**Figure 1:**
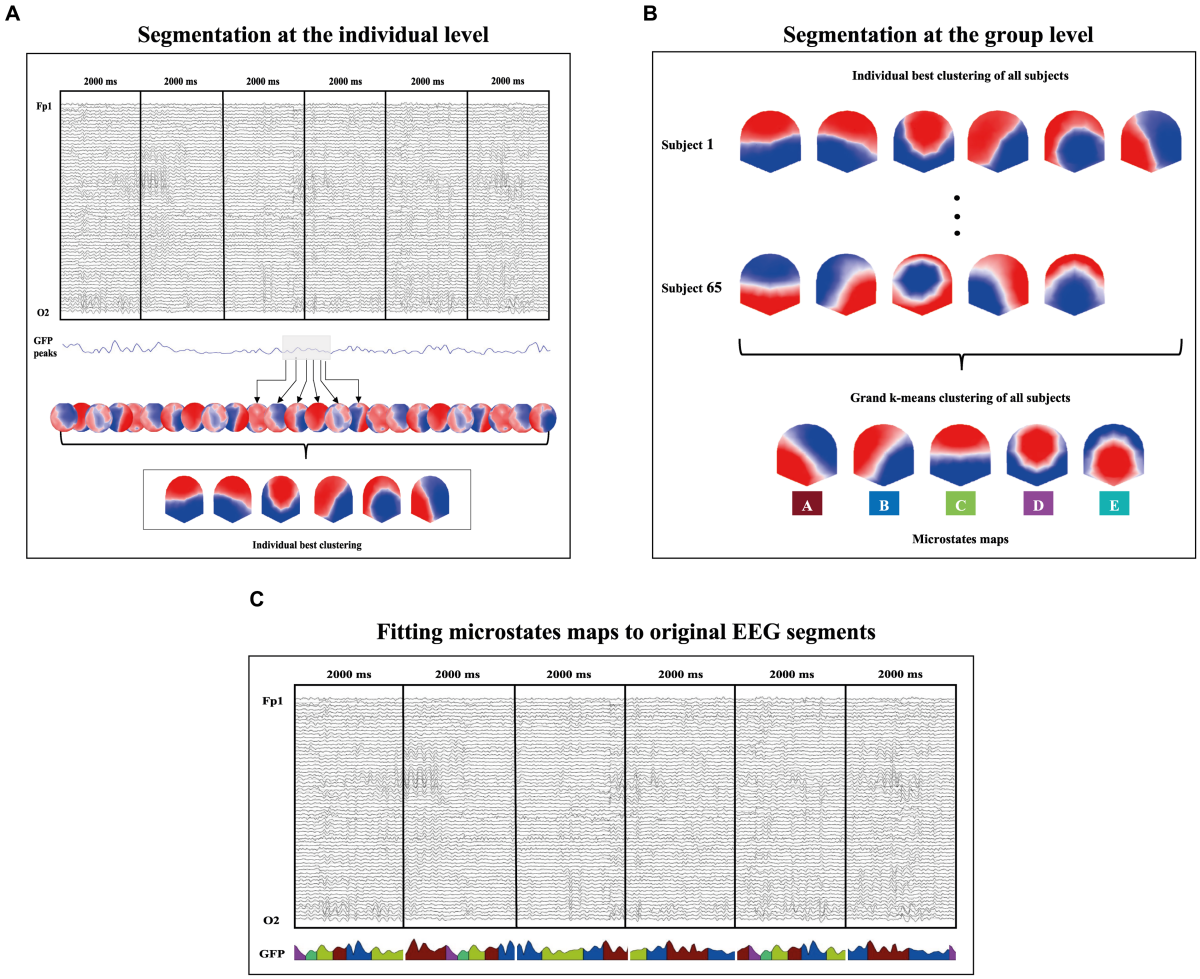
Graphical illustration of the microstate analysis procedure. **(A)** A first k-means clustering is applied to identify the most representative microstates of each participant. This step is done on the topographies at each GFP peak of the signal as illustrated by the black arrows. **(B)** A second k-means clustering is applied to the best representative microstates of each participant to identify the most representative microstates topographies across all participants. This procedure led to the identification of five microstate class explaining 86.44% of the global variance. **(C)** These microstates were then fit back to the original prestimulus periods that best correlates with the individual maps (*r >* 0.5) of each subject. This procedure allowed the computation of the microstates’ temporal parameters (GEV, GFP, Mean duration, Time coverage, and Frequency of occurrence).

The EEG microstates analyses (Figure 1) were performed with the Cartool software by Denis Brunet (brainmapping.unige.ch/cartool) and followed the steps proposed by Michel and Koenig (2018). This analysis is based on the Global Field Power (GFP) which represents a measure of the scalp field strength and corresponds to the standard deviation of all electrodes at a given period (Skrandies, 1990). The datasets containing the prestimulus epochs of each participant were submitted to a first k-mean clustering to identify the most representative microstates of each participant. A second k-mean clustering was applied to the best representative microstates of each participant to identify the most representative microstates topographies across all participants. These microstates topographies were then fit back to the original prestimulus periods that best correlates with the individual maps (*r* > 0.5) of each subject. Each map that did not reach this threshold remained unlabelled. Temporal smoothing parameters: windows half size = 3; strength (Besag factor) =10 were fixed as well as rejection of small segments shorter than 3 time frames (23.44 ms). This fitting process allowed the computation of the following microstates temporal parameters for each microstate class (A, B, C, D and E): the Global Explained Variance (GEV, the sum of the explained variances of each microstate weighted by the global field power), the mean GFP, the mean duration (average time in milliseconds covered by a given microstate), the time coverage (percentage of the time covered by a given microstate), and the frequency of occurrence (mean number of the occurrence of a distinct microstate within 1 s).

### 2.6. Statistical analyses

#### 2.6.1. Relationship between prestimulus microstates and VO_2_max

To explore the association between prestimulus microstates and VO_2_max, we conducted a correlation analysis between the temporal parameters of each microstate (GEV, GFP, mean duration, time coverage, and frequency of occurrence) and VO_2_max. Given the multiple correlations performed for each microstate (i.e., five correlations), we adjusted the alpha level to 0.01 to control for the potential increase of type 1 error.

#### 2.6.2. Relationship between prestimulus microstates and response times

For statistical analyses, microstates temporal parameters were averaged each ten trials. We used a multilevel mixed effects model to investigate the linear association between prestimulus microstates parameters and response times. Microstates class, response times and interaction between microstates class and response times were set as fixed effect factors. A random effect was set to represent the between-participant variability. Microstates temporal parameters were set as the dependent variable. Indeed, the temporal parameters were concatenated across all prestimulus epochs for each microstate and were used as dependent variables (GEV, GFP, Mean duration, Time coverage and Frequency of occurrence). We used restricted maximum likelihood estimation to estimate the parameters of the models and degrees of freedom were calculated by Satterthwaite method.

Results are reported as type III *F* tests for omnibus effects. In addition, parameter estimates for simplified models with significant fixed effects are provided. For simple effects, models’ parameter estimates are reported. Considering the use of five dependent variables (microstate parameters), the alpha of all type III *F* tests in our mixed effects models was corrected and set at 0.010 to account for increased type 1 error. Further, to account for additional simple tests of effects for each microstate class, we set the alpha at 0.002 (five microstate parameters and five microstates).

## 3. Results

### 3.1. Behavioral results

The multiple linear regression showed that the VO_2_max significantly predicted the response times in the PVT (*t* = 2.51; *β* = 0.35; 95% CI [0.64, 0.07]; *p* = 0.015) while age, BMI and Beacke total score did not predict the response times. For details on the behavioral results, refer to the study of Di Muccio et al. (2022).

### 3.2. Microstates analysis

Based on the meta-criterion proposed by the Cartool software (for details, see Custo et al., 2017), the k-means clustering identified five microstate classes that explained the best the variance across all participants (86.44%). These microstate classes were labeled A, B, C, D and E (Figure 1B) in accordance with previous literature (Michel and Koenig, 2018; Zanesco et al., 2020a).

#### 3.2.1. Relationship between prestimulus microstates and VO_2_max

**Figure 2:**
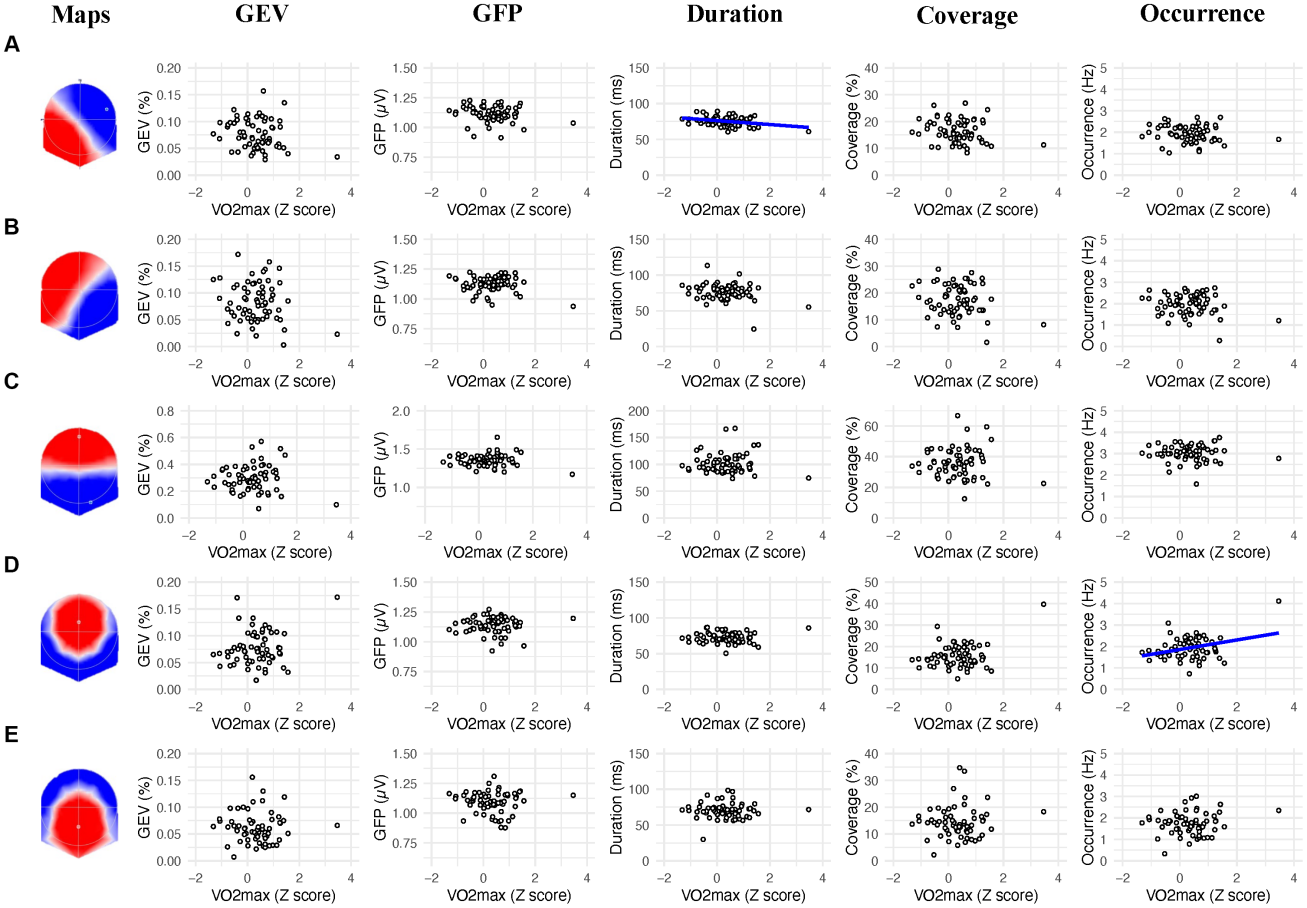
Linear association between the 2s prestimulus microstates parameters and the VO_2_max of participants for each microstate class (**A−E**). Blue regression lines indicate significant correlations (*p* < 0.01).

We found a significant negative correlation between the mean duration of the microstate A and the VO_2_max (*r* = −0.320; *p* = 0.009), showing that lower duration of the microstate A was associated with higher VO_2_max (Figure 2, column 3). We also found a significant positive correlation between the frequency of occurrence of the microstate D and the VO_2_max (*r* = 0.326; *p* = 0.008), showing that higher frequency of occurrence of the microstate D was associated with higher VO_2_max (Figure 2, column 5).

#### 3.2.2. Prestimulus microstates and response times

We computed a multilevel mixed effects model to investigate the linear association between prestimulus microstates parameters and the response times of participants. Parameter estimates of the models are reported in Table 2.

**Table 2:** Parameter estimates of the analyses of response times and EEG microstates parameters.

	Estimate (SE)				
Parameter	GEV (%)	GFP (μV)	Duration (ms)	Coverage (%)	Occurrence (Hz)
Fixed effects					
Intercept	0.120 (0.001)*	1.162 (0.004)*	79.222 (0.521)*	20.000 (0.080)*	2.122 (0.022)*
Microstate A	−0.219 (0.002)*	−0.239 (0.004)*	−26.415 (0.545)*	−19.550 (0.253)*	−1.088 (0.018)*
Microstate B	−0.212 (0.002)*	−0.241 (0.004)*	−24.954 (0.545)*	−18.500 (0.253)*	−1.049 (0.018)*
Microstate D	−0.219 (0.002)*	−0.217 (0.004)*	−29.405 (0.545)*	−20.230 (0.253)*	−1.092 (0.018)*
Microstate E	−0.228 (0.002)*	−0.266 (0.004)*	−31.516 (0.545)*	−21.790 (0.253)*	−1.290 (0.018)*
RT	0.119 (0.001)	0.005 (0.043)	−2.536 (5.262)	5.785 (1.637)	0.053 (0.183)
RT* A	−0.239 (0.042)*	−0.006 (0.091)	−38.053 (11.155)*	−20.930 (5.177)*	0.138 (0.376)
RT* B	−0.351 (0.042)*	−0.500 (0.091)*	−73.408 (11.155)*	−39.370 (5.177)*	−1.700 (0.376)*
RT* D	−0.407 (0.042)*	−0.402 (0.091)*	−67.711 (11.155)*	−50.290 (5.177)*	−2.697 (0.376)*
RT* E	−0.403 (0.042)*	−0.527 (0.091)*	−82.072 (11.155)*	−44.460 (5.177)*	−1.823 (0.376)*
Participants (*N*)	65	65	65	65	65
Observations	7,790	7,790	7,790	7,790	7,790

Restricted maximum likelihood estimates are provided from mixed effects models analyzing the relationship between response times and pre-stimulus microstates parameters. Microstate C and response times served as the reference. The standard errors (SE) are reported in parentheses. * *p* < 0.01.

##### 3.2.2.1. Global explained variance

For associations between the GEV and response times, we found a significant main effect of microstate class (*F* (4, 7,716) = 4528.229, *p* < 0.0001), no main effect of response times and a significant interaction between microstate class and response times (*F* (4, 7,716) = 32.573, *p* < 0.0001). Simple effects showed that the GEV of the maps C (*t* = 8.936; *β* = 0.291; 95% CI [0.227, 0.355]; *p* < 0.0001), D (*t* = −3.577; *β* = −0.117; 95% CI [−0.181, −0.053]; *p* = 0.0004) and E (*t* = −3.429; *β* = −0.112; 95% CI [−0.176, −0.048]; *p* = 0.0006) significantly predicted the response times. Higher GEV of the map C was related to slower response times while higher GEV of the maps D and E were related to faster response times.

##### 3.2.2.2. Mean global field power

For associations between the GFP and response times, we found a significant main effect of microstate class (*F* (4, 7,716) = 1202.068, *p* < 0.0001), no main effect of response times and a significant interaction between microstate class and response times (*F* (4, 7,716) = 16.720, *p* < 0.0001). Simple effects showed that the GEV of the maps A (*t* = 3.987; *β* = 0.286; 95% CI [0.145, 0.426]; *p* < 0.0001), B (*t* = −2.914; *β* = −0.209; 95% CI [−0.349, −0.068]; *p* = 0.0036), C (*t* = 4.071; *β* = 0.292; 95% CI [0.151, 0.432]; *p* < 0.0001) and E (*t* = −3.283; *β* = −0.235; 95% CI [−0.376, −0.095]; *p* = 0.0010) significantly predicted the response times. Higher GFP of the maps A and C was related to slower response times while higher GFP of the maps B and E was related to faster response times.

##### 3.2.2.3. Mean duration

For associations between the mean duration and response times, we found a significant main effect of microstate class (*F* (4, 7,716) = 1105.322, *p* < 0.0001), no main effect of response times and a significant interaction between microstate class and response times (*F* (4, 7,716) = 18.113, *p* < 0.0001). Simple effects showed that the mean duration of the maps C (*t* = 5.648; *β* = 49.710; 95% CI [32.459, 66.967]; *p* < 0.0001) and E (*t* = −3.677; *β* = −32.360; 95% CI [−49.613, −15.105]; *p* = 0.0002) significantly predicted the response times. Higher mean duration of the map C was related to slower response times while higher mean duration of the map E was related to faster response times.

##### 3.2.2.4. Time coverage

For associations between the time coverage and response times, we found a significant main effect of microstate class (*F* (4, 7,780) = 2550.630, *p* < 0.0001), no main effect of response times and a significant interaction between microstate class and response times (*F* (4, 7,780) = 31.450, *p* < 0.0001). Simple effects showed that the time coverage of the maps C (*t* = 8.471; *β* = 31.009; 95% CI [23.833, 38.185]; *p* < 0.0001), D (*t* = −5.267; *β* = −19.280; 95% CI [−26.455, −12.104]; *p* < 0.0001) and E (*t* = −3.673; *β* = −13.446; 95% CI [−20.622, −6.270]; *p* = 0.0002) significantly predicted the response times. Higher time coverage of the map C was related to slower response times while higher time coverage of the maps D and E was related to faster response times.

##### 3.2.2.5. Frequency of occurrence

For associations between the frequency of occurrence and response times, we found a significant main effect of microstate class (*F* (4, 7,716) = 1565.522, *p* < 0.0001), no main effect of response times and a significant interaction between microstate class and response times (*F* (4, 7,716) = 21.600, *p* < 0.0001). Simple effects showed that the time coverage of the maps A (*t* = 4.692; *β* = 1.407; 95% CI [0.819, 1.995]; *p* < 0.0001), C (*t* = 4.231; *β* = 1.269; 95% CI [0.681, 1.857]; *p* < 0.0001) and D (*t* = −4.761; *β* = −1.428; 95% CI [−2.016, −0.840]; *p* < 0.0001) significantly predicted the response times. Higher frequency of occurrence of the maps A and C was related to slower response times while higher frequency of occurrence of the map D was related to faster response times.

## 4. Discussion

**Figure 3:**
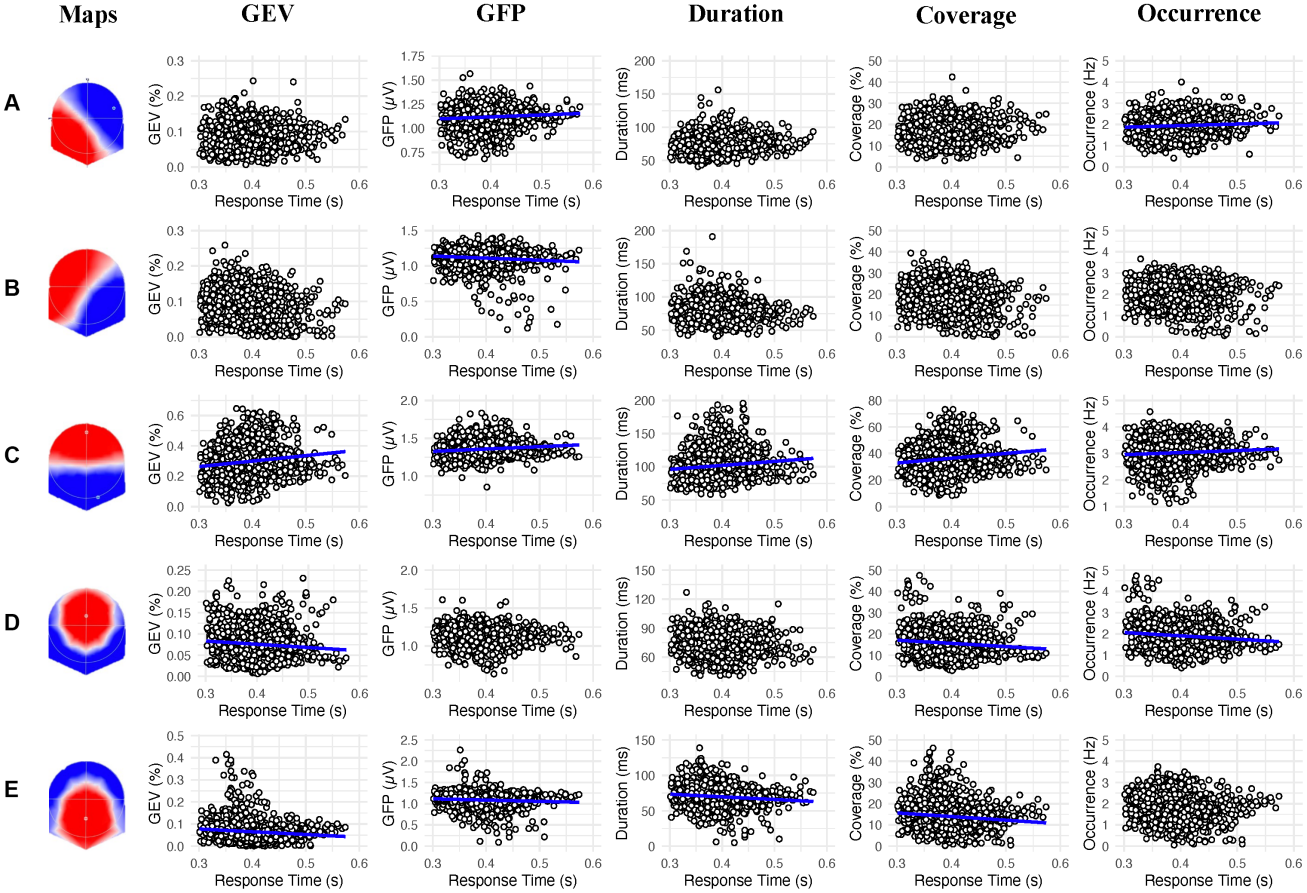
Linear association between the 2s prestimulus microstates parameters and the response times in the psychomotor vigilance task for each microstate class (**A−E**). Blue regression lines indicate significant associations (*p* < 0.002).

In the present study, we examined the relationship between prestimulus electrocortical brain activity, CRF, and response times in a sustained attention task. We hypothesized that individuals with higher CRF levels and faster response times would exhibit a lower prevalence for microstate C, suggesting a lower activation of DMN, and a higher prevalence for microstate D, suggesting a higher activation of DAN which could reflect their faster response times in the PVT. Results indicate that a lower duration of the microstates A and a higher occurrence of the microstate D correlate with higher CRF levels (Figure 2). However, a relationship between microstate C and CRF was not observed. Concerning the response times, we found that a higher GFP and occurrence of the microstate A and a higher GEV, GFP, Duration, Coverage, and occurrence of the microstate C were associated with slower response times. On the other hand, a higher GFP of the microstate B, a higher GEV, coverage and occurrence of the microstate D, as well as a higher GEV, GFP, duration and coverage of the microstate E were associated with faster response times (Figure 3).

### 4.1. Role of microstates A and C

We found that a shorter duration of the microstate A was associated with higher CRF whereas a higher GFP and occurrence of this microstate were both linked to slower response times. This microstate has been associated with the auditory resting state network (Michel and Koenig, 2018). The mechanisms by which this microstate is less prevalent in high-fit than low-fit participants and before faster response times during the PVT have yet to be fully elucidated. A possible explanation for this result could be related to the vigilance state. Indeed, a previous study showed that higher levels of vigilance during resting states correlate with a lower prevalence of microstate A (Krylova et al., 2020). In the context of the PVT, it is likely that the lower prevalence of microstate A exhibited by participants with higher CRF is due to a higher vigilance state during prestimulus periods. However, this interpretation remains speculative and further research is needed to confirm the link between microstate A and the level of vigilance.

Contrary to our hypothesis, there was no association between the prevalences of microstate C and CRF. Because (i) this microstate has been associated with the DMN (Custo et al., 2017) and (ii) an increased prestimulus prevalence of the microstate C has been associated with poorer sustained attention performance (Zanesco et al., 2020a), we expected it to be less activated in participants with a high CRF compared to those with a low CRF. Although our results support those of Zanesco et al. (2020a) by showing an increased GEV, GFP, duration, coverage, and occurrence of microstate C with slower response times, this relationship does not seem to depend on the CRF level.

### 4.2. Role of microstates B, D and E

We did not find any relationship between the prevalence of the microstate B and CRF. However, the relationship between higher GFP of the microstate B and faster response times corroborate the findings of previous work in this field. This microstate has been linked to the visual resting state network (Britz et al., 2010; Custo et al., 2017). Interestingly, it has been shown that the prevalence of microstate B is higher when the eyes are open compared to when they are closed (Seitzman et al., 2017). Furthermore, Bréchet et al. (2019) found a higher prevalence of the microstate B during a mentally memory task as compared to a mental arithmetic task and a resting condition. The authors interpreted the role of the microstate B as related to a memory reconstruction system. In our task, it can be suggested that the microstate B could be involved in the maintenance of the task goal in the working memory throughout the task.

In agreement with our hypotheses, we found that higher occurrence of microstate D correlated with higher CRF levels. In addition, higher GEV, coverage and occurrence of the microstate D was associated with faster response times. This microstate has been associated with the DAN (Britz et al., 2010; Custo et al., 2017) and is congruent with previous studies that show higher prevalence of microstate D during cognitive tasks (Seitzman et al., 2017; Bréchet et al., 2019; Kim et al., 2021). For instance, these works showed an increased prevalence of microstate D during mental arithmetic tasks compared to resting states. In addition, this microstate positively correlated with vigilance levels at rest (Krylova et al., 2020). Accordingly, it can be hypothesized that increased occurrence of microstate D in high-fit participants reflect stronger attentional states during prestimulus periods. Therefore, the prevalence of microstate D may explain improved performances observed in fit participants during PVT compared to low-fit participants (Di Muccio et al., 2022).

Finally, the result of this study did not show any relationship between the prevalence of the microstate E and CRF. However, we found an association between higher GEV, GFP, duration and coverage of the microstate E and faster response times. This result is in accordance with the study of Zanesco et al. (2020a) that found a lower prevalence of this microstate when participants reported to feel mentally out of the task. This microstate is more rarely detected in the literature because generally only four microstates are used in the clustering analysis. According to Custo et al. (2017), since microstates C and E are spatially correlated, they often collapse to microstate C when the clustering analysis is limited to four microstates. It is however common for the microstate E (also named C′ or F depending on the study) to appear when the analysis allows the emergence of more than four microstates (Custo et al., 2017; Bréchet et al., 2019; Zanesco et al., 2020a,b, 2021; Férat et al., 2021, 2022; Artoni et al., 2022). The intra-cerebral sources of this microstate have been localized in the dorsal anterior cingulate, the superior and middle frontal gyrus as well as the insula (Custo et al., 2017). Interestingly, these regions are part of the salience network described by Uddin et al. (2019). The functional role of the salient network is to identify relevant cues according to the context such as environmental stimuli or internal sensation and memory. It is therefore possible to hypothesize that a higher prevalence of microstate E prior to a stimulus apparition is associated with a greater readiness to respond to this stimulus.

There were limitations in this study. First, the cross-sectional design limited the ability of the study to demonstrate a causal relationship between CRF and prestimulus brain activity during sustained attention tasks. Therefore, future studies should investigate this relationship through randomized control trials. Additionally, laboratory-based sustained attention tasks, such as PVT, are poorly applicable to real-world situations. Consequently, researchers should investigate prestimulus brain dynamics within ecological contexts with a mixture of targets and distractors to better understand CRF’s role in sustained attention tasks.

## 5. Conclusion

In conclusion, this is the first study to investigate the relationship between prestimulus brain activity and CRF during a sustained attention task. The results show that prestimulus electrocortical activity correlates with CRF in the PVT. For individuals with higher CRF, prestimulus brain activity is characterized by a decreased prevalence of microstates A (associated with increased vigilance levels) combined with an increased prevalence of microstate D (associated with increased dorsal attentional network activity). In addition, an increased prevalence of microstate A was associated with slower response times in the psychomotor vigilance task, while an increased prevalence of microstate D was linked to faster response times. Accordingly, it can be suggested that this electrocortical dynamic typically exhibited by high-fit individuals allows them to allocate their attentional resources efficiently.

## Data availability statement

The raw data supporting the conclusions of this article will be made available by the authors, without undue reservation.

## Ethics statement

The studies involving human participants were reviewed and approved by Cantonal Ethics Committee for Human Research (Vaud, Switzerland; protocol 2018-02107). The patients/participants provided their written informed consent to participate in this study.

## Author contributions

FM: conceptualization, methodology, software, formal analysis, investigation, data curation, writing – original draft, visualization, and project administration. MS: writing – review and editing. CB: conceptualization, methodology, resources, supervision, and project administration. PR: conceptualization methodology, software, formal analysis, and writing – review and editing. JB: conceptualization, methodology, formal analysis, resources, writing – review and editing, supervision, and project administration. All authors contributed to the article and approved the submitted version.

## Funding

The open-access publication fees were supported by the University of Lausanne, Lausanne, Switzerland. Open access funding was provided by the University of Lausanne.

## Conflict of interest

The authors declare that the research was conducted in the absence of any commercial or financial relationships that could be construed as a potential conflict of interest.

## Publisher’s note

All claims expressed in this article are solely those of the authors and do not necessarily represent those of their affiliated organizations, or those of the publisher, the editors and the reviewers. Any product that may be evaluated in this article, or claim that may be made by its manufacturer, is not guaranteed or endorsed by the publisher.
